# Medical Geography and Endemic Diseases
*Traité de Géographie et de Statistique Médicales et des Maladies Endemiques, comprenant la météorologie et la géologie médicales, les lois statistiques de la population et de la mortalité, la distribution géographique des maladies, et la pathologie comparée des races humaines. Par J. Ch. M. Boudin, Médecin-en-Chef de l'Hôpital Militaire du Roule; Officier de la Légion d'Honneur. Avec neuf cartes et tableaux. Tomes ii, 8vo. Bailliére, Regent Street.


**Published:** 1857-10

**Authors:** B. Daniel


### MEDICAL GEOGRAPHY AND ENDEMIC DISEASES.*

A SCIENCE is called exact when all its details can be referred to first principles, and when, starting from first principles, we are able to educe their necessary consequences in detail. Thus, in mathematics, we arrive at the knowledge of unknown things by accurate reasoning on certain known axioms. Every line starts from a point. Every series begins with a fixed number. With this idea the Pythagoreans endeavoured to translate the law of nature into the law of numbers. Statisticians are doing the same thing. The progress of science has been from the simple to the complex: from mathematics to physics, from physics to astronomy and chemistry, from these to meteorology, and to the foundation of physiology and pathology. Thus, of all human studies, there is nothing more comprehensive or more elaborate than that which treats of the phenomena of living beings; and as man is of living beings the archetype, the highest known living organisation, so is that problem the most difficult to solve which seeks to render an account of the relation he bears to the external world, to explain how he acts, and how he is acted upon. From time immemorial the inquisitive nature of man has sought to unravel the mystery of his physical being. The biblical word and proper name *Adam* stands for *earth, blood, man.* It has been left to modern science in the hands of a Liebig to show by chemical demonstration how these are mutually convertible. Hippocrates explained life by regarding it as a product of nature; and there has been no lack of ingenuity in the invention of fictitious principles which were to afford the key to the solution of vital actions. The ancients and moderns have vied with each other in their haste to render premature solutions of the hidden springs of nature. We have had the *pneuma* of Athenæus, the *arche* of Paracelsus and Van Helmont, and the *soul* of Stahl. The material system of Leucippus and Democritus has been revived by modern materialists, and life has been explained as a *property* of matter. Electricity, caloric, fermentation and mechanical equilibrium have each in turn been held up by their respective admirers as the efficient causes of all

* Traité de Géographie et de Statistique Médicales et des Maladies Endemiques, comprenant la météorologie et la géologie médicales, les lois statistiques de la population et de la mortalité, la distribution géographique des maladies, et la pathologie comparée des races humaines. Par J. Ch. M. BOUDIN, Médecin-en-Chef de l'Hôpital Militaire du Roule; Officier de la Légion d'Honneur. Avec neuf cartes et tableaux. Tomes ii, 8vo. Baillière, Regent Street.

vital phenomena. Bacon, with his "non fingo hypotheses", started a new era. Philosophers no longer were to be allowed to amuse themselves with inventing inexplicable theories to explain inexplicable things. There was the book of nature laid open before them. There was the "Mene, Mene, Tekel Upharsin" for those who had the wish to interpret. An age of physical inquiry and registration of facts was inaugurated. When these had been recorded by numerous observers, it was found that their isolation was an embarrassment, and men sought to tie them together. Newton suggested *gravitation* as a bond of common union among a certain class of facts; and this also was a fact which was found to be a general law applicable to the heavens as well as to the earth. But Newton never set up gravitation of itself as a cause of any phenomena. The solution of its essential nature he never sought to unravel.

The system of experimental inquiry in physics led to the adoption of the same method in the pursuit of other natural sciences; and Cuvier showed, by his observations on general anatomy, how to distinguish, by a small bone of the foot, the peculiar characteristics of the animal to which it belonged. In his history of the progress of natural sciences, he observes, "that in the period between 1789 and 1808 natural history began to be recognised in its true character—namely, a science the object of which is to apply the general laws of mechanics, physics and chemistry, to the explanation of the particular phenomena presented by various bodies in nature."* It may be said that the philosophic inquirer into the origin of disease is now bending all his efforts to trace the connection which exists between diseases and their source, whether these proceed from within or without. The causes of all diseases must be sought either in the innate constitution of our bodies, or in the external action of the agents by which we are surrounded. It follows then, that by an accurate examination of the peculiar effects produced by climatic relations, we may be able to assign to temperature, to atmosphere, to humidity, to locality, and to other physical agencies, their individual and specific actions. When the observation of facts shall be sufficiently extensive, let us hope that another Newton may come on the stage to solve the problem of generalisation of the laws of life, but let us avoid narrow contracted views. Hasty hypotheses and generalisations have been the bane of science.

In the meantime, all experience and knowledge may be made immediately applicable for the alleviation of disease, and for

* Cuvier, Histoire des progrès des Sciences Naturelles.

preventing such catastrophes as those which attended the French expedition to St. Domingo in the beginning of the present century, the Walcheren invasion during the epidemic season in 1809, and the advance into Russia by the French in 1812. A knowledge of medical geography would have taken cognisance of yellow fever, of marsh fever, and of the effects of congelation. We have taken these instances from the book we have in review; and the errors which have prevailed with regard to acclimatisation, as well as the importance of the subject, are thus referred to by the author, who says, in the Introduction :—

"It is the question of acclimatisation which must rule the selection of colonies, and the choice of troops destined to serve in countries more or less distant from the mother country. But the strangest errors have prevailed on the subject of acclimatisation, the inconveniences of which have sometimes been exaggerated and sometimes extenuated. Cassini thought no animal could live above 4,767 *mètres** above the level of the sea, whilst observation has taught us that men inhabit regions close upon 4,800 *mètres* of the same measure of altitude. Aeronauts have ascended upwards of 7,000 *mètres*. According to Boerhaave, no animal provided with lungs is able to live in an atmosphere the temperature of which equals that of its own blood, whilst the indigenous inhabitant of certain countries of the globe enjoys perfect health with the thermometer (cent.) at 47°† in the shade and 70°‡ in the sun. On the other hand, a celebrated geographer, Malte Brun, affirms that 'under every climate, the nerves, the muscles and the vessels, in dilating or contracting, soon take upon themselves the habitual condition which is most suitable to the degree of heat or of cold to which the body is exposed.'" (p. xxxvii.)

Dr. Boudin is of opinion that to a certain degree the human race is capable of adapting itself to any climate, but he is not a believer in the power of acclimatisation being without limits. He questions whether the negro is capable of preserving his integrity of intellectual and physical organisation, and of perpetuating his race without the tropics. He also gives a table showing the gradual extinction of race which is taking place among the negroes in the British West Indies, and a tabular view of an official report of the relation which exists between the births and the deaths, in which the latter are in excess. He quotes a remarkable report of Col. Tulloch, who says: "That before a century has elapsed, the negro race will

* The mètre is equal to 39·37 English inches.

† $116\frac{3}{5}°$ F. ($47° \text{ C} \times \frac{9}{5} = 84\frac{3}{5} + 32 = 116\frac{3}{5}°$ F.)

‡ 158° F. ($70° \text{ C} \times \frac{5}{9} = 126 + 32 = 158°$ F.)

almost have disappeared from the British colonies in the West Indies."

In respect of the power of the European constitution to adapt itself to a hot climate much misapprehension has prevailed.

"For a long time many governments, somewhat in accordance with medical theories, hoped, by a protracted residence in hot climates, that the European garrisons would diminish their ratio of mortality; but the practical application of this theory was followed by the most disastrous results. On the other hand, the system of a triennial renewal of these garrisons, adopted by the English government, already shows, by statistical returns, how prudent and how beneficial to the health of the troops this change of system has been." (vol. i, p. 40.)

The proportionate ratio of mortality which exists between the English and the native troops in India is a subject of great importance:—

"During the period from 1825 to 1844 the mean annual mortality of India has been, in the province of Bombay, 50 per 1000 men for the English troops, and 12 only for indigenous troops. In Bengal, 73 for British troops; 17 for the natrive troops. In Madras, 38 for the British; and 20 for the native troops. In Sierra Leone, the annual mortality, which in British troops amounts to 483 deaths to 1000 men, is reduced to 30 per 1000 among the negro troops. But the most curious and interesting observation is, perhaps, that which has been made during a series of years in the island of Ceylon, where the comparative ratio of mortality has been noted among five different races of which the troops are composed. Thus—

| | Ann. Deaths per 1000. |
|---|---|
| Native troops of Bengal and Madras.............. | 12 |
| Troops recruited on the coast of Ceylon .......... | 23 |
| Malays ...................................... | 24 |
| Negro troops.................................. | 50 |
| English troops ............................... | 69 |

Thus race and nationality show themselves of the highest importance in the consideration of recruiting for foreign stations; not alone as an object of humanity, but of the highest consequence in political and financial economy." (*Ibid.*)

The high rate of mortality which is thus shown to exist in British troops in India, in proportion to that which prevails amongst the native troops, being nearly quadruple, must present itself as a subject of the greatest importance at the present juncture of Indian affairs. With such an experience before us, it will scarcely be deemed practicable to hold India by the continued presence of large bodies of British troops, among whom a natural process of extinction is continually going on. Without doubt the present mutiny will be suppressed by the energy and vigour of European material organisation, discipline and courage; but a policy which will make the native feel that it is his best interest to serve us is the only one that can ever preserve India as the subject of Great Britain.

A table is given by the author, in which will be shown the increasing depopulation which exists among European colonists in Egypt and in other parts of Africa.

"According to reports presented by the French Minister of War, the mortality of the French population, which in France is about 24 deaths in 1000, and during the cholera of 1849 never attained to 28 in 1000, ascends in Algiers to the following figures:

| | Deaths to 1000 Inhabitants. |
|---|---|
| In 1848 to more than | 41 |
| 1849 | 101 |
| 1850 | 70 |
| 1851 | 64 |
| 1852 | 55 |
| 1853 | 47 |

"In 1854, that is, during the last year in which government had published its report, the number of deaths of the European population to the number of births were as 7025 is to 611." (p. 38.)

The following passage, which alludes to the power of acclimatisation with which certain races are endowed, is worthy of quotation:

"There are types of races which have a wonderful power of adaptation to the changes of climate, while others are scarce able to support the least change. Among the former, we may cite the Jew and the Gypsy. The Jew at the present moment is to be found in every part of the world: in Europe, from Norway to Gibraltar; in Africa, from Algiers to the Cape of Good Hope; in Asia, from Cochin to the Caucasus; from Jaffa to Pekin; in America, he is to be met with from Montevideo to Quebec; for the last fifty years he has invaded Australia; and has given proofs of his power of acclimatisation under the tropics where people of European origin have constantly failed to perpetuate themselves. In relation to altitude, although he seldom inhabits the mountains, for his tendencies are usually industrial or commercial, yet there is nothing to make us suppose that he possesses any physical incompatibility for residence in elevated localities. On the other hand, he has lived for many ages, and lives on still on the only point of the globe, the valley of the Jordan, which is situated more than 400 *mètres below* the level of the sea, and where it is doubtful whether any European would ever suceeed in propagating his race. Finally, wherever the Jewish race has been studied up to the present time, it has been found to submit to statistical laws of births, deaths, and proportions of sex, differing completely from those which govern the nationalities among whom they reside. Assuredly, so unexpected a fact, one so contrary to reasoning, is not one of the least interesting of the facts which medical geography has demonstrated to us."

Speaking of diseases in a geographical and historical point of view, the author says :—

"The diseases of humanity have differed according to time and space. History describes to us diseases of former times unknown at the present, and scourges unknown to ancient times devastate modern populations. Thus Pliny the naturalist observed nearly eighteen centuries ago: 'Id ipsum mirabile videtur, alios in nobis morbos desinere, alios durare"; and fourteen centuries later Sydenham has it: 'Sicuti alii morbi jam olim exstitere qui vel jam ceciderunt penitus, vel ætate saltem pene confecti exolevere, et rarissimi comparent; ita qui nunc regnant morbi, aliquando demum intercident, novis cedentes speciebus, de quibus nos ne minimum quidem hariolari valemus.' When account is taken of the absence of sporadic cases of plague in the East during the last fourteen years, and of its gradual diminution for the last two centuries, it appears more than probable that this disease is becoming gradually extinct." (p. 41.)

There can be little doubt but that the type of disease must have much changed even in our own times in England. It must be within the memory of most practitioners how much the use of the lancet has fallen off, as well as the employment of depletory medicines. Mercury, tartar emetic, and opium, formed the tripod on which every practical man took his stand. The antiphlogistic diet was in vogue. Disease was regarded as a raging fire, which was to be extinguished by all means, and at all hazards. Sometimes the patient succumbed, and sometimes recovered. Of one thing we may be quite sure, that if the same system of antiphlogistic treatment were adopted at the present time, such heroic treatment, as it was called (facetiously perhaps), would be uniformly fatal. Are we to say then that our forefathers were less skilful or less observant than ourselves? Certainly not. But we must conclude, what history teaches us, that the type of disease has changed, and what might have been a proper treatment fifty years ago, would be a very dangerous one now. *Tempora mutantur.* The drunken *bonhommie,* which prevailed in the good time when George was king, might be a very jolly life for the robust bacchanals of those days, who were able to enjoy themselves without much deterioration either of their mental or their physical vigour. It is not unreasonable to imagine that depletion was the best thing for constitutions capable of such rough usage.

We pass on more particularly to a notice of the author's remarks on Medical Geography. He observes:

"In regard to the distribution of diseases according to space, it belongs to the province of medical geography; and its study is of the highest interest, even in the most practical point of view. It may be sufficient for the medical man whose practice is circumscribed within a definite locality, to know the particular topographical nature of its diseases; but it is not so with the physician who resides in a centre, having constant relations with all parts of the world, and even less so with the medical officers of the army and navy, who are constantly required to change their residence. For these latter, it is their duty to be conversant with the diseases of all parts of the globe to which they may be destined, for upon this knowledge the success of an expedition or the safety of an army may depend.

"Like plants, some of which are disseminated and found in every part of the globe, whilst others are, as it were, endemically circumscribed to certain defined localities, so the diseases of the human family, some of them are universally distributed over the surface of the earth, while others are found restricted to certain zones and localities. Like plants, diseases have their *habitats*, their *stations*, their geographical limits. The northern limit of the cholera in Europe is found to be Archangel, about 64° north latitude. It has as yet spared from its attacks Iceland, Greenland, and Siberia; in America it has ascended up to Canada, and it has attained its southern boundary in 21° south latitude. The Cape of Good Hope and Australia have as yet escaped this scourge.

"The limit of 'marsh fevers' in the old continent may be represented by the isothermal curve of 15° centig. The north of Scotland, the Hebrides, Orcades, Shetland, Faroe Islands, and Iceland thus escape. In the southern hemisphere, the domain of marsh fever does not even reach the isothermal line of 15° centig. Yellow fever has never passed the 48° of north latitude, nor the 27° of southern latitude. Its home is represented by the shores of the Gulf of Mexico and of the Caribbean Sea (*la mer des Antilles*) and along the American coast of the Pacific Ocean. Pellagra is to be found between 42° and 46° north latitude. The 'tubercle of Aleppo' (*le bouton d'Alep*) between 33° and 38°; the bereberi, between 16° and 20° north.

"In respect of geographical longitude, similar limitations prevail. Thus in the Scandinavian peninsula, the *radesyge** is to be found eastward, and the *spedalskhed* to the westward of the mountains. A disease, *verugas*,† is restricted to the western declivity of the Andes in Peru. Yellow fever has only been observed between Livorno and Acapulco. The plague has its eastern limitation in a

---

* A cutaneous affection.

† This is also a tubercular cutaneous disease, analogous to the elephantiasis of the Greeks. *Spedalsk*=a leper.

line drawn from the Gulf of Mexico and extending to the Caspian Sea." (p. 42.)

The degree of elevation exerts its specific action on the character of disease. Thus the author observes:—

"Verugas is only found between 600 and 1,600 *mètres* above the level of the sea. In Mexico, the yellow fever never ascends higher than 924 *mètres*. Cretinism, which in South America is to be observed above 4,000 *mètres* of elevation, in Piedmont never rises higher than 2,000 *mètres*, and in Switzerland not higher than 1,000 *metres*. Of 10,000 inhabitants of Piedmont, there are found 35 cretins in the mountains, and only four in the plains; 100 suffer from goître in the mountains, and 16 only in the plains. Sometimes the influence of elevation is represented by a simple modification of the form of disease. For instance, marsh fever, as it recedes from the equator or from the summer season, loses its type of continuity, and becomes more and more intermittent; and so also in hot and marshy countries, in proportion to elevation, do these fevers pass gradually from continued to the rarest intermittents, thus representing in effect a perfect stratification and graduation of types. There are diseases strictly local, confined within circumscribed limits: thus *verugas*, in Peru; *pinta*, in Mexico; *caak*, in Nubia; *plica*, in Poland; *tubercle of Zibau* (*bouton de Zibau*), in Algiers; hydatids of the liver, in Iceland. Other affections, although not strictly localised, yet present themselves with an exceptional frequency: such are tænia, in Abyssinia; cataract, in the Bay of Biaffra; croup, in certain parts of Sweden; the trismus of new-born babes, in the island of Westmannö; pemphigus, in Ireland; and *bicho*, in Brazil.

"Some countries are remarkable for the rarity or absence of certain diseases: thus pellagra is wanting in Sicily and Sardinia; gout is scarcely known in Peru, in Brazil, or in Nubia; phthisis, very rare in the archipelago of Viti, is almost unknown in Iceland, in the Faroe islands, and in the steppes of the Kirghis; vesical calculi are rare in Pisa, Madrid, and Guiana; hæmorrhoids are never observed in Nubia; scrofula, rare in the Faroe islands and in the steppes of the Kirghis, is completely absent from Iceland. Obesity is very rare in North America.

"The nature of the soil seems in many cases to have an immediate connexion with the character of prevalent diseases. Cholera is found to have a marked predilection for a tertiary and a clayey soil; goître is to be found specially on a soil of chalk metamorphosed by magnesia, while adjoining granite and oolithic districts are exempt.

"Such is the intimate connexion (*solidarité*) between the soil and certain diseases, that modification of the former is followed by a corresponding change in pathological demonstration. On many points of the United States and of Switzerland, the disappearance of marsh fever by desiccation of the soil has been followed closely by the appearance, or by the multiplication of pulmonary phthisis."*

Remarkable evidence is given by the author of the preventative operation of a seafaring life with respect to phthisis. (p. 45.)

"It has been shown that the deaths from consumption in the British army are, in the line, 8·9 in 1,000 men, in the guards 12·5 in 1,000 men; whilst in the British navy, in the years from 1830 to 1836 inclusive, the deaths from phthisis have been in

| | Men per 1000. |
|---|---|
| The United Kingdom | 1·7 |
| Mediterranean | 1·9 |
| Missions and correspondence | 1·9 |
| West coast of Africa and the Cape of Good Hope | 1·7 |
| East Indies | 1·4 |
| West Indies and North America | 1·9 |
| South America | 1·7 |
| Average | 1·7 |

The effect of temperature in the maintenance or production of disease is shown by the facts that "yellow fever requires a temperature of 20° centigr. (68° Fahr.) for its development in an epidemic form, and that the epidemic form of plague disappears from Egypt when the temperature approaches 28° centigr. (82° Fah.) On the other hand, typhus is found to prevail in winter and spring, and tends to disappear in summer. It has been remarked that the stokers of steam vessels have a strong predisposition to attacks of yellow fever and to dry colic." The author professes to have remarked, during a mission he undertook in Provence in 1856, an analogous disposition to typhus among the stokers and cooks arriving in steam vessels from the Crimea. The hepatitis of hot climates has mostly been attributed to excessive heat; but tables of the mortality of the army at different colonies prove that locality as well as heat has its peculiar influences. The effect of temperature on the rate of mortality caused by phthisis is worthy of particular notice. It is found that among the troops the maximum of deaths from this disease prevail in the United Kingdom of Great Britain, and that the mortality not only diminishes as the climate becomes warmer in Jamaica, West Indies, Bermudas, Mauritius, and Ceylon, but that an equal diminution takes place as the climate becomes colder, *e. g.* Nova Scotia and Canada. The author observes:

"Perhaps one of the most curious results obtained from our re-

* Drake, Principal Diseases of the Interior Valley of North America, as they appear in the Caucasian, Indian, African, and Esquimaux varieties of its population.

searches into medical geography, is the increasing diminution of the ravages of consumption as we proceed northward from the 44th degree north latitude in America and from the 58th in Europe. This law is shown in England by an almost entire absence of pulmonary phthisis in the north of Norway, in the Faroe islands, and in Iceland. Of 100 deaths from every cause, there are from phthisis: in London, 18; Edinburgh, 11·9; Leith, 10·3; Aberdeen, 6·2." (p. 48.)

Certain localities present diseases occasioned by particular parasitical animals.

"Thus in Iceland, hydatids of the liver attack a seventh part of the population. In Egypt, the *distoma hæmatobium*, which is the real cause of the epidemic vesical catarrh and of the calculous affections which prevail there; tænia prevails all along the African coast from the Mediterranean shores to the Cape of Good Hope. In Geneva, a fourth of the population either have had, or will have, the bothriocephalus; while at Zurich, the tænia solium is alone found. In the east of Europe, the Vistula separates the two species: on the right bank is found the bothriocephalus; on the left, the tænia solium.

"In regard even to the mode of committing suicide, geographical influences prevail: thus the Frenchman blows out his brains in the proportion of three or four to one in comparison with the Englishman, the Saxon, the Norwegian, or the Dane; he drowns himself in proportion of two or three to one of the English. The people of Germanic origin have a preference for death by hanging"

The space which we have already devoted to the work before us, which is of two octavo volumes of above 1,200 pages, is already too much extended to allow of any more quotations from the abundant collection of interesting and novel facts which they contain. We have done our duty in calling the attention of our readers to a work which from beginning to end we have found most attractive. We have been embarrassed by the richness of the materials how to cull the choicest of them for our readers. We agree with the author that—

"The geographical distribution of diseases is a subject of interest to science, to practical medicine, to public hygiene, and to political economy. It throws a light upon the influences of locality, of race and of nationality, in the production of disease; it guides the physician in the choice of a climate most suitable to special disease; it regulates the institution of quarantine, and the enlistment of armies."

It not only does these things, but it enlarges our experience in extending the domain of recognised facts; and when these shall be sufficiently known, we may hope, by careful consideration and analysis, to come to an exact appreciation of the influence of physical causes upon the human body in health and disease. It is enough for the practical purpose of the mariner that the compass points North and South; and so, for the practical physician, a knowledge of the beneficial or the injurious influence of physical agents may be made a practical science. The mariner adapts his sails to the prevailing breezes, and Mahomet, because the mountain would not come to him, went to the mountain; so the physician, who cannot command the powers of nature, may, nevertheless, so place his patient under influences most favourable either for his recovery from disease, or for the development and perfectibility of the human constitution.

We cannot close this review without paying a tribute to the enterprise of the publisher, M. J. B. Baillière, under whose auspices this work has appeared. We may, however, observe, that both scientific and charlatanic medicine are equally indebted to this enterprise. The tact which perceives the scientific medical necessities of the day is not less than that which perceives the ready market afforded by a depraved public taste and morality for works especially addressed to credulity and sensuality. The publication of such works is as much a stain upon the profession as the work before us is an ornament to it.

*B. Daniel.*

